# Ultra Wideband Radar Cross Section Reduction by using non-Resonant Unit Cells

**DOI:** 10.1038/s41598-020-64362-0

**Published:** 2020-05-14

**Authors:** Morteza Nadi, Seyed Hassan Sedighy, Mohamad Khalaj-Amirhosseini

**Affiliations:** 1 School of Electrical Engineering, Iran University of Science and Technology, Tehran, Iran; 2 School of New Technologies, Iran University of Science and Technology, Tehran, Iran

**Keywords:** Electrical and electronic engineering, Electronics, photonics and device physics

## Abstract

A general approach is proposed to design ultra-wideband radar cross section reduction (RCSR) metasurface by using non-resonant unit cells in chessboard arrangement. The proposed miniaturized artificial magnetic conductor unit cell is composed of two stacked non-resonant patches separated from one another by thin dielectric substrates. The genetic optimization algorithm is used to optimize the unit cell design parameters and obtain wide 10-dB RCSR bandwidth. The proposed approach is performed to design three different RCSR metasurfaces, ideal, ROGERS and low cost. The low cost RCSR metasurface composed of low cost commercially available FR-4 substrate is fabricated and tested which reduces RCS more than 10-dB from 5.22 GHz to 30.85 GHz, 142% fractional bandwidth. This metasurface has significantly wider RCSR bandwidth rather than the state of the art references as well as low cost, simple and light weight structure which facilities its practical applications.

## Introduction

Within the last decade, the radar stealth technology has been developed rapidly and resulted in new methods for radar cross section reduction (RCSR). There are various technical methods for RCSR such as shaping and absorbing materials. The shaping method usually affects the aerodynamics and mechanical aspects of the object which limit its benefits. In the absorption method, the resonance absorbers such as Dallenbach layer are placed on a conducting plane to converting the incident electromagnetic energy into heat^1^. The Salisbury screens are used as absorber, also which employs a resistive sheet above a metallic surface with a distance of a quarter wavelengths. However, the low bandwidth and high thickness of these layers limit their applications specially in the moving platforms^2^.

The metasurfaces are considered in the early published papers for RCSR, also which can achieve thin effective RCSR surface with wide bandwidth performance. Metasurfaces are two dimensional metamaterials composed of subwavelength periodic array elements. Several design approaches have been reported to reduce the RCS with metasurfaces^3–10^. The chessboard like surface arrangement with PEC (reflecting incident waves with a 180° phase change) and PMC tiles (reflecting incident waves without phase change) has been proposed in^3^ to achieve destructive reflective phase and RCSR, consequently. Two different AMC tiles instead of PEC-AMC ones in the chessboard like arrangement have been proposed to enhance the RCSR bandwidth, also. For example, two different AMC tiles formed by saltire arrows and four E-shaped unit cell arrays were employed in^5^ to enhance 10-dB RCSR bandwidth more than 85%. The uneven layered coding AMC tiles were presented in^6^ which consists of square ring unit cells and resulted 10-dB RCSR from 6.2 to 25.7 GHz. Using more than one layer AMC metasurface can be employed to achieve 109% RCS reduction as discussed in^11^. The polarization conversion metasurface has been proposed for wideband RCS reduction, also. For example, a metasurface consists of double heads arrow unit cell with its 90°, 180° and 270° rotated ones has been proposed in^7^ to create the destructive interferences cancellation and RCSR, consequently from 9 to 40 GHz (126.5%) for normally TM- and TE- polarized incident waves.

**Figure 1:**
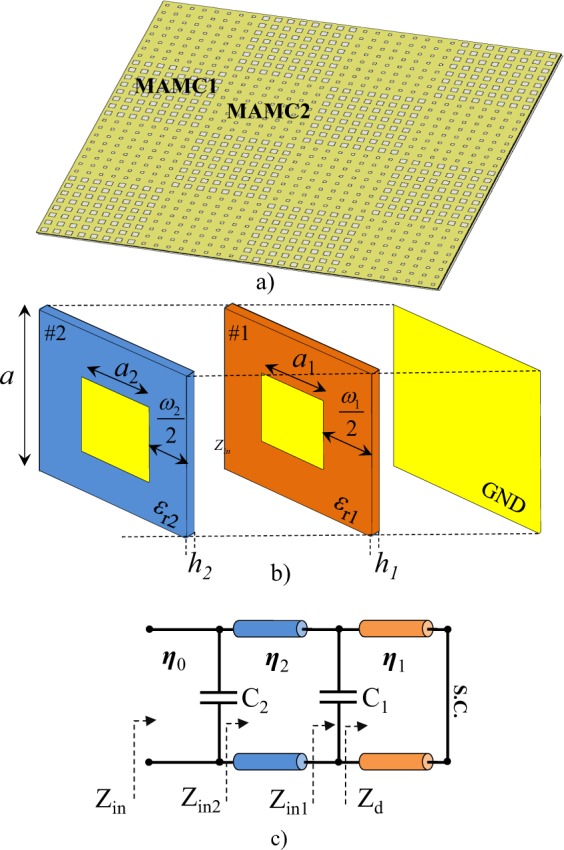
(**a**) The chessboard like proposed RCSR metasurface structure includes two different MAMC tiles (**b**) The composition of each MAMC (**c**) MAMC equivalent circuit model.

In this paper, a new technique is proposed to achieve ultra-wideband RCSR by using miniaturized artificial magnetic conductor (MAMC). The MAMCs are periodic highly-miniaturized non-resonant unit cells composed of stacked non-resonant patches separated from one another by thin dielectric substrates^12^. The non-resonance behavior of this unit cell achieves more linearity in the reflection phase response versus frequency rather than the resonant ones. These unit cells can be designed to achieve wideband 180° ± 37° destructive reflection phase cancellation in chessboard arrangement and RCSR, consequently as shown in Fig. 1(a). For this purpose, the design parameters of the unit cell are optimized with genetic algorithm (GA) to achieve the widest RCSR bandwidth. This design method is employed to design three different RCSR surfaces: *ideal metasurface* without any implementation consideration, *ROGERS metasurface* used commercial available RF substrates which have closest electrical constant achieved in *ideal metasurface*, and *low cost metasurface* used low cost commercial available FR-4 substrate. The 10-dB RCS reduction bandwidth of the proposed structure is about 150% for *ideal metasurface*, 133% for *ROGERS metasurface* and 142% for *low cost metasurface*. The *low cost metasurface* is fabricated and tested as representative case. The 10-dB RCSR bandwidth of the proposed surface is more than 98% for TM- and more than 51% for TE- polarization up to 40° oblique incident angles.

The proposed structure has significantly wider RCSR bandwidth rather than the state of the art refs. ^4,6,7,9–11,13–15^, as well as low cost, simple and light weight structure which facilities its practical applications.

## Design Procedure

### MAMC unit cell Modeling

Figure 1(b) shows the proposed MAMC unit cell which is composed of capacitive patches and the thin dielectric substrates modeled with parallel capacitors and transmission lines, respectively. The ground plane is modeled as short load (S.C.) at one side, also. The equivalent circuit model of this unit cell is also shown in Fig. 1(c). The capacitance values at each layer can be calculated by mapping between the equivalent circuit model and physical parameters of the unit cell. The effective capacitance in each layer (C_1_, C_2_) can be approximated by the following closed form expression^16^

1 $${C}_{i}=\frac{2a{\varepsilon }_{0}{\varepsilon }_{re}^{(i)}}{\pi }Ln\left(\frac{1}{\sin \left(\frac{\pi {\omega }_{i}}{2a}\right)}\right)$$

where *C*_*i*_ (*i* = 1, 2) is the effective capacitance of the sub-wavelength patch layer, *a* is the period of the unit cell, $\frac{{\omega }_{i}}{2}$(${\omega }_{i}=a-{a}_{i}$) is the gap between two adjacent capacitive patches on each layer, and ${\varepsilon }_{re}^{i}$ is the effective dielectric constant of the medium that the capacitive layers are embedded.

The input impedance of a lossy grounded dielectric slab at normal incidence can be expressed as

2 $${Z}_{d}=\frac{{\eta }_{{0}}}{\sqrt{{\varepsilon {\prime} }_{r1}+j{\varepsilon {\prime\prime} }_{r1}}}\,{\tanh }({k}_{0}\sqrt{{\varepsilon }_{r1}^{{\prime} }+j{\varepsilon }_{r1}^{{\prime\prime} }}{h}_{1})$$

where *η*_0_ is the free space characteristic impedance, *k*_0_ is the free space propagation constant, and *h*_1_ is the first layer dielectric substrate thickness. The input impedance of the layer1 (Z_in1_) is resulted from parallel connection between two complex impedances; 1/jωC_1_ and Z_d_ which is related to the thickness and dielectric constant of the first layer and the first patch width, a_1_. This impedance can be considered as the load for the second layer where the input impedance Z_in2_ achieved by using the transmission line theory. This impedance (Z_in2_) is paralleled with C_2_ and simply achieves Z_in_ which is related to patches widths (*a*_1,2_), substrates constants (*ε*_1,2_) and heights (*h*_1,2_). Therefore the reflection coefficient of this unit cell in periodic arrangement can be expressed as

3 $$\varGamma =\frac{{Z}_{in}-{\eta }_{0}}{{Z}_{in}+{\eta }_{0}}$$

where the phase of Γ achieves the unit cell reflection phase.

For an ideal lossless structure, this reflection coefficient is achieved where the real part of the input impedance is zero (Re(Z_in_) ≅ 0). Therefore, the MAMC reflection phase is related to the patch length, thickness, and dielectric constant of the both layers which can be approximated as:

4 $${\rm{\angle }}\varGamma =\pi -2\arctan \left(\frac{\text{Im}({Z}_{in}({\varepsilon }_{r1,2},{h}_{1,2},{a}_{1,2}))}{{\eta }_{0}}\right)$$

To achieve wideband RCS reduction, two different unit cells should be designed with 180° ± 37° phase difference in the bandwidth. To evaluate the proposed MAMC behavior versus a_1_ and a_2_ variations, a unit cell with Rogers 4003 (ε_r_ = 3.38 and tanδ = 0.003) substrate for both layers and 1.6 mm and 3.2 mm thicknesses for the first and second layers is designed and simulated by CST Microwave Studio software. The phase response of the designed MAMC are extracted by applying periodic boundary conditions and exciting the unit cell with Floquet port.

**Figure 2:**
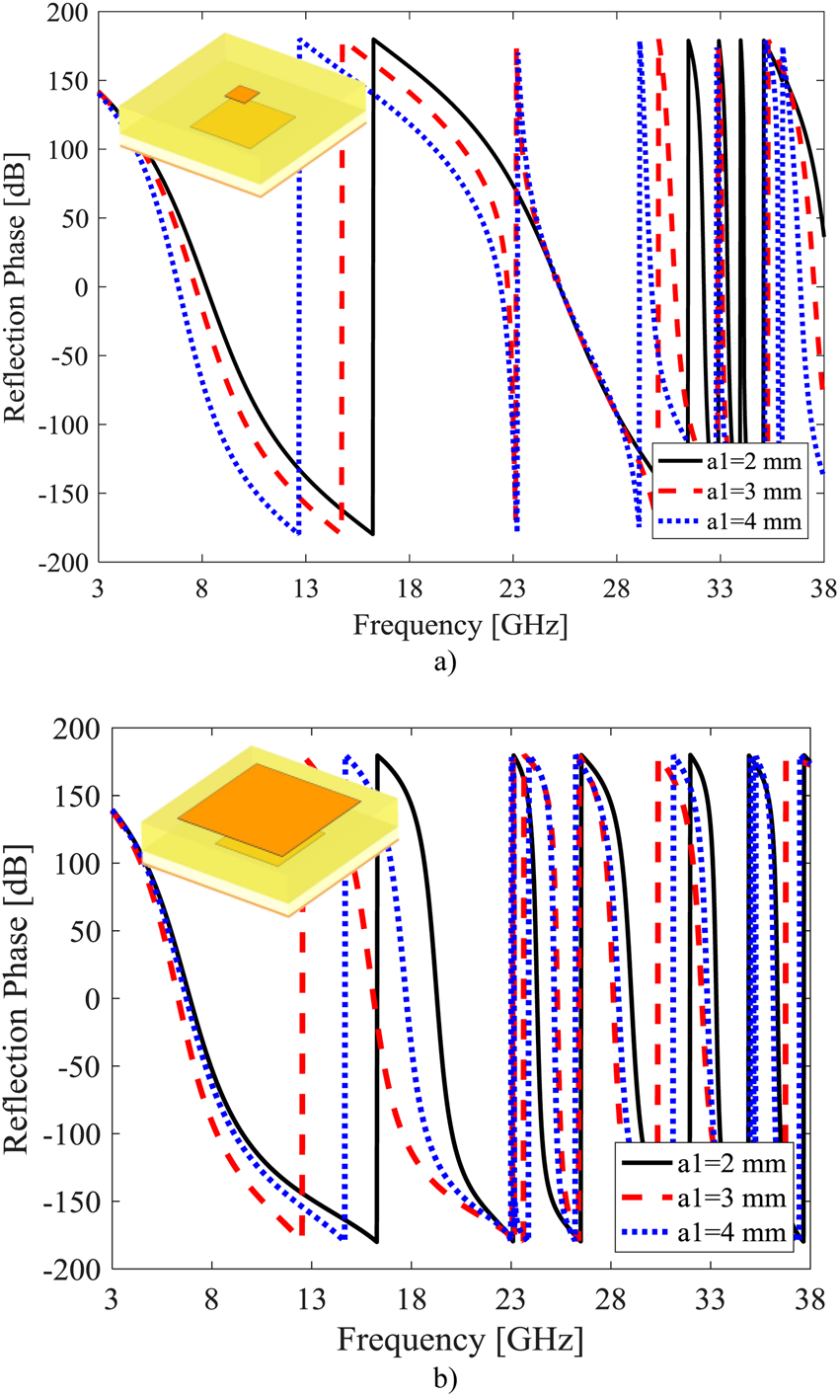
Simulation results of reflection phase for different a1 (**a**) a2 = 1 mm. (**b**) a_2_ = 5 mm.

**Figure 3:**
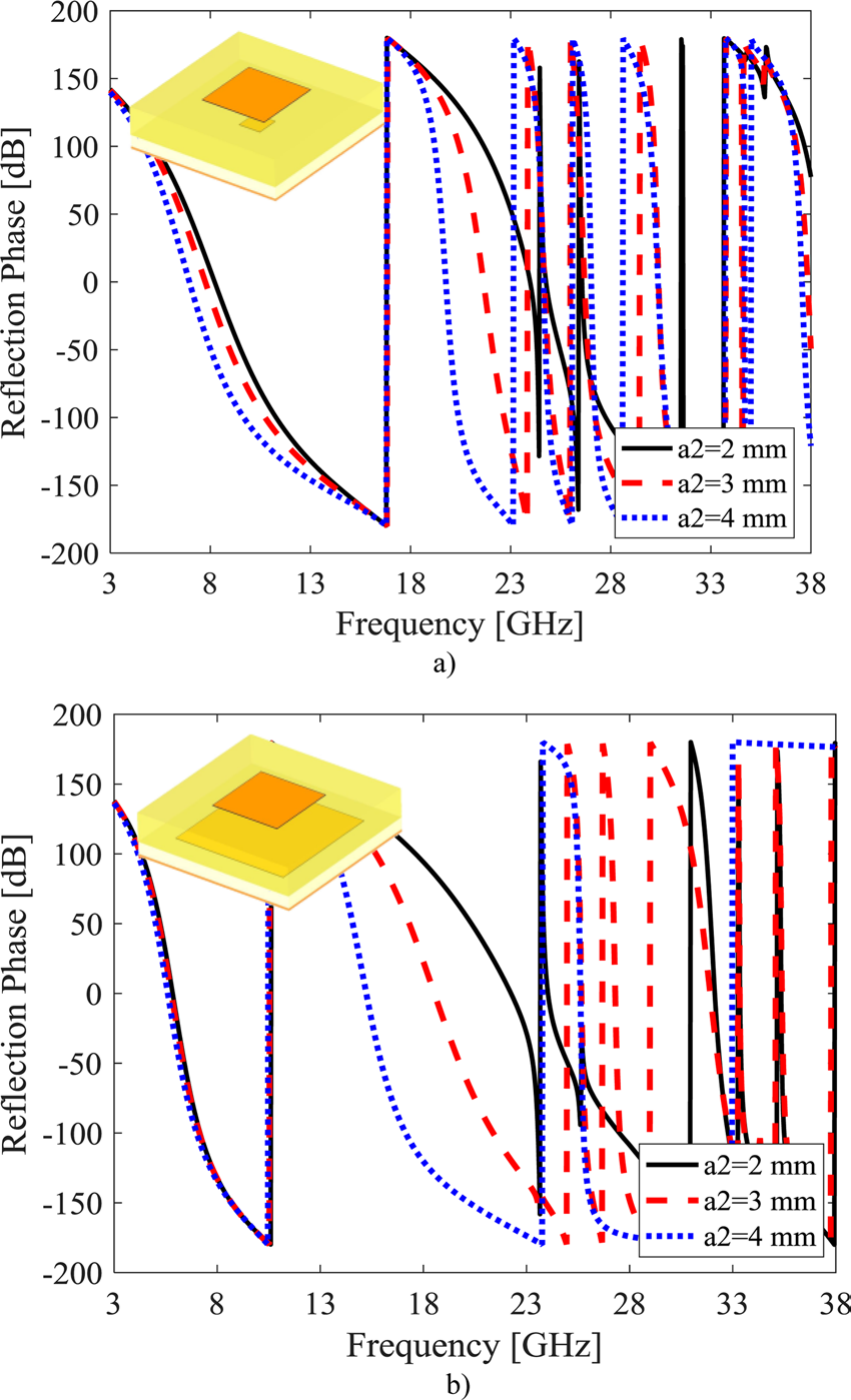
Simulation results of reflection phase for different a2 (**a**) a1 = 1 mm. (**b**) a_2_ = 5 mm.

The reflection phase of this unit cell versus frequency is shown for different a_1_ values where a_2_ = 1 mm (upper patch is smaller than lower one) in Fig. 2(a) and a_2_ = 5 mm (upper patch is larger than lower patch) in Fig. 2(b). In similar way, the reflection phase of this unit cell versus frequency is shown for different a_2_ values where a_1_ = 1 mm (lower patch is smaller than upper patch) in Fig. 3(a) and a_1_ = 5 mm (lower patch is larger than upper patch) in Fig. 3(b). It can be seen, the dimension variation of the upper patch (a_2_) and lower patch (a_1_) lead to change in the slope of reflection phase response as well as its exchange from −180° to +180°. Therefore, one can achieve wide bandwidth 180° ± 37° phase difference by proper selection of these parameters. In the next subsection, the GA optimization approach is used to achieve these values, properly.

### Optimization

**Figure 4:**
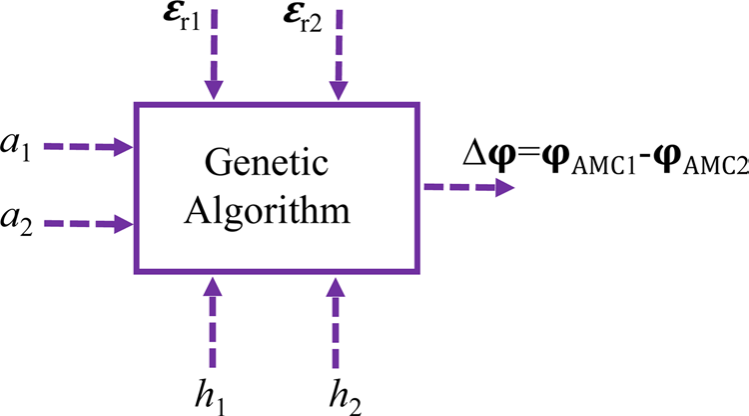
Genetic algorithm module for optimization.

Here, the GA algorithm is used to obtain 180° ± 37° phase difference between two unit cells in wide bandwidth, assuming that the unit cell period is fixed in each MAMC. The unit cell design parameters such as length (a_1_ and a_2_), thicknesses (h_1_ and h_2_) and dielectric constant of each layer (ε_1_ and ε_2_) are the input of Matlab optimization code, as illustrated in Fig. 4. The GA tries to find the best parameters of these two MAMC unit cells which satisfy the desired phase difference 180° − 37° < Δφ_i_ < 180° + 37° in the specified frequency bandwidth, where i∈ [f_min_, f_max_].

**Figure 5:**
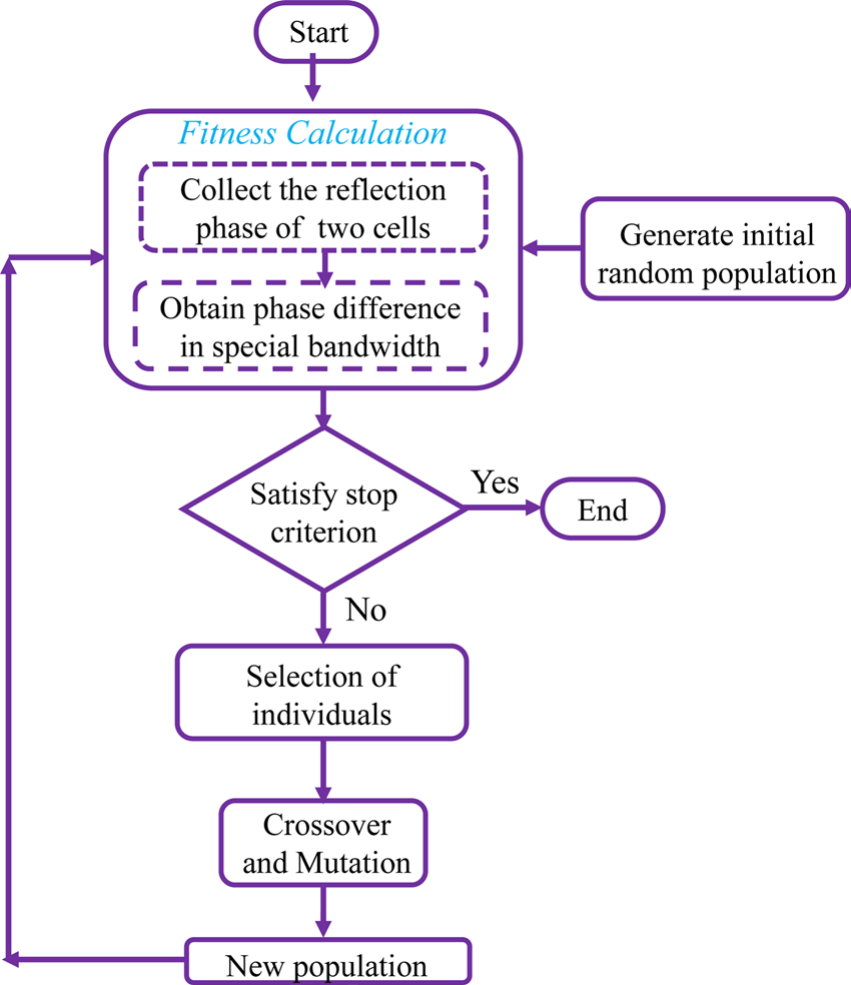
GA Flowchart employed to search for the optimal unit cell design parameters.

In fact, the GA optimization minimizes the cost function value subject to the upper (180° + 37°) and lower (180° − 37°) violation values for 10-dB RCSR as follow

4a $${Violatio}{{n}}_{{1}}=\mathop{\sum }\limits_{k=0}^{N}{\max }\left(\frac{\varDelta {\varphi }_{({f}_{start}+k\times {f}_{step})}}{{180}+{37}}-{1},\,{0}\right)$$

4b $${Violatio}{{n}}_{{2}}=\mathop{\sum }\limits_{k=0}^{N}{\max }\left(1-\frac{\varDelta {\varphi }_{({f}_{start}+k\times {f}_{step})}}{{180}-{37}},\,{0}\right)$$

4c $${Cos}\,{t}=\mathop{\sum }\limits_{k={1}}^{{2}}{\alpha }_{k}\times {({Violatio}{{n}}_{{k}})}^{{2}}$$

where *N* = (*f*_max_ - *f*_min_)/*f*_step_ and α_k_ is the weighting coefficient and their values should emphasize the relative importance of each term in the cost function. The optimization procedure is iterated until the cost function is neared to zero or the maximum iterations are met. The design flowchart is shown in Fig. 5, also. The optimization outputs are the optimum design values of two different unit cells which satisfy the 180° ± 37° phase difference criteria in wide bandwidth.

### Design Examples

In this section, three examples are proposed to design MAMC unit cell based on the proposed optimized approach to achieve 180° ± 37° phase differences at maximum frequency bandwidth. In the first example, the substrate availability is not considered and the optimized design values are extracted ideally (*ideal metasurface*). In the second example, the closest available commercial RF substrates extracted in the *ideal metasurface* are considered in the design approach (*ROGERS metasurface*). Finally, the low cost commercial available FR4 substrate is used to achieve *low cost metasurface* in the third example. The values of *f*_min_ and *f*_max_ are considered as 5 GHz and 35 GHz, respectively in the optimization process.

### Case I: Ideal metasurface

**Figure 6:**
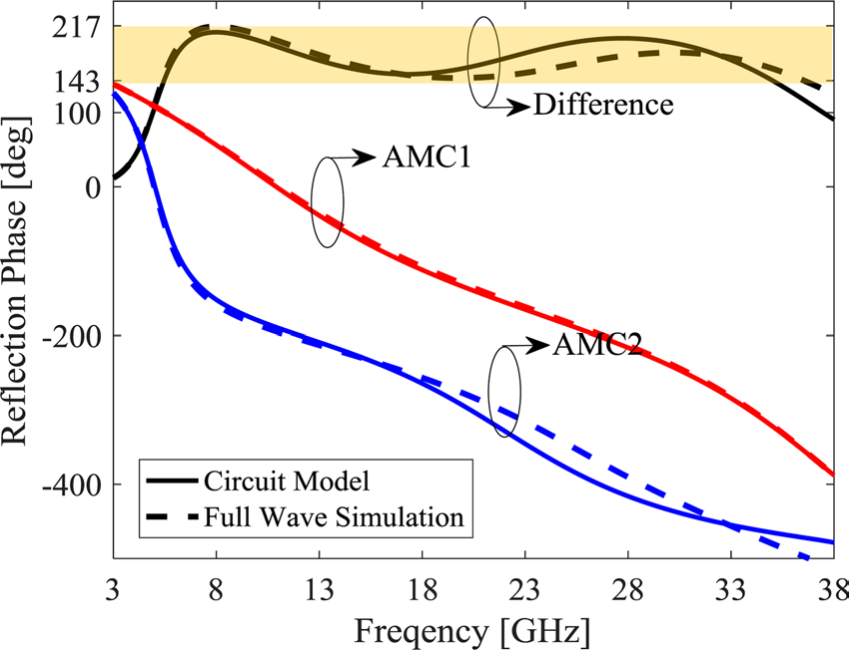
Reflection phase from two MAMC unit cells in *Ideal metasurface*.

The most bandwidth of 180° ± 37° phase difference can be achieved by using two different unit cells with a_1_ = 5.85 mm and a_2_ = 2.7 mm for the MAMC1 and a_1_ = 1.17 mm and a_2_ = 0.4 mm for the MAMC2 one. The optimized dielectric constant and thickness values are ε_r1_ = 1, h_1_ = 3.9 mm for the first layer and ε_r2_ = 1.7, h_2_ = 1.69 mm for the second layer. The unit cell dimension is 6 × 6 mm^2^, also. Figure 6 depicts the reflection coefficient of these unit cells (MAMC1 and MAMC2) where the solid lines demonstrates the results achieved from the circuit model proposed in Fig. 1 and the dashed line depicts the full wave simulation results. The good agreement between these two results prove the proposed model, also. The 180° ± 37° phase difference is achieved from 5.462 GHz to 35.02 GHz (146% 10-dB RCSR bandwidth) in the equivalent circuit model and 5.37 GHz to 37.5 GHz in the full wave simulation, corresponding to a 150% fractional bandwidth in this case.

### Case II: ROGERS metasurface

**Figure 7:**
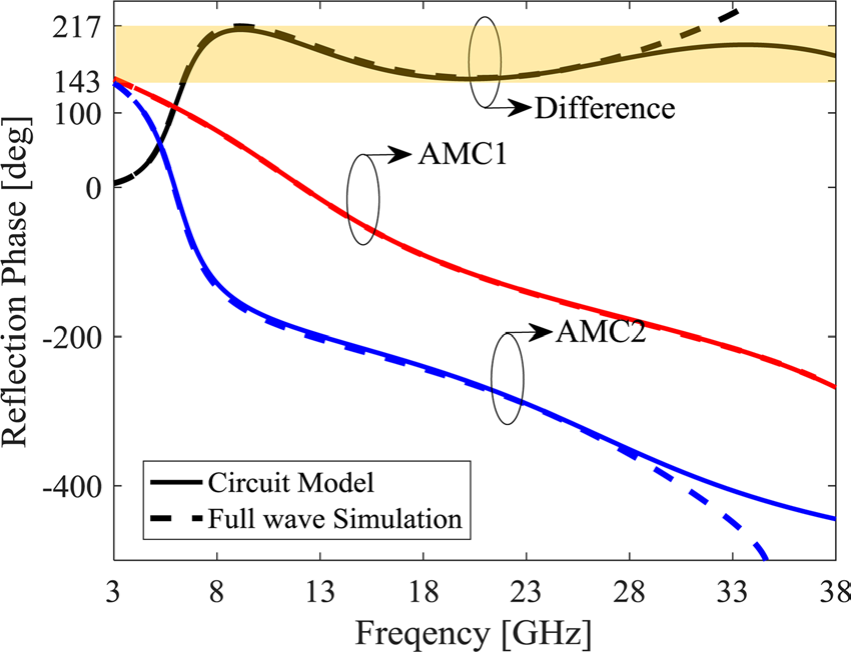
Reflection phase from two MAMC unit cells for ROGERS metasurface.

The first layer relative permittivity in *ideal metasurface* is ε_r_ = 1 which can be implemented by air gap simply with the corresponding height (h_1_ = 3 mm), but the second layer with ε_r_ = 1.7 and h_2_ = 1.69 mm is not commercially available. Therefore the closest commercially available substrate to the optimized values (ε_r2_ = 1.7, h_2_ = 1.69), Rogers 5880 with ε_r_ = 2.2 and tanδ = 0.0009 specifications is used as the second layers but with 1.6 mm thickness. Therefore, a_1_ and a_2_ for both MAMC unit cells and *h*_1_ are obtained from the GA optimization process. The reflection phase of these two optimized MAMC unit cells are shown in Fig. 7 versus frequency. The optimized values are a_1_ = 0.4 mm and a_2_ = 0.4 mm for MAMC1 and a_1_ = 6.5 mm and a_2_ = 2 mm for MAMC2. It can be seen that 180° ± 37° phase difference is achieved from 6.425 GHz to 41.14 GHz (146% fractional bandwidth) in the circuits model and 6.37 GHz to 31.72 GHz in the full wave simulation, corresponding to a 133% fractional band width. The lower phase difference bandwidth in the circuit model is due to the higher mode propagation at high frequency which is not considered in the model. After the optimization, the unit cell periodicity is slightly tuned to achieve wider phase difference bandwidth which results to a = 7 mm.

### Case III: Low Cost metasurface

**Figure 8:**
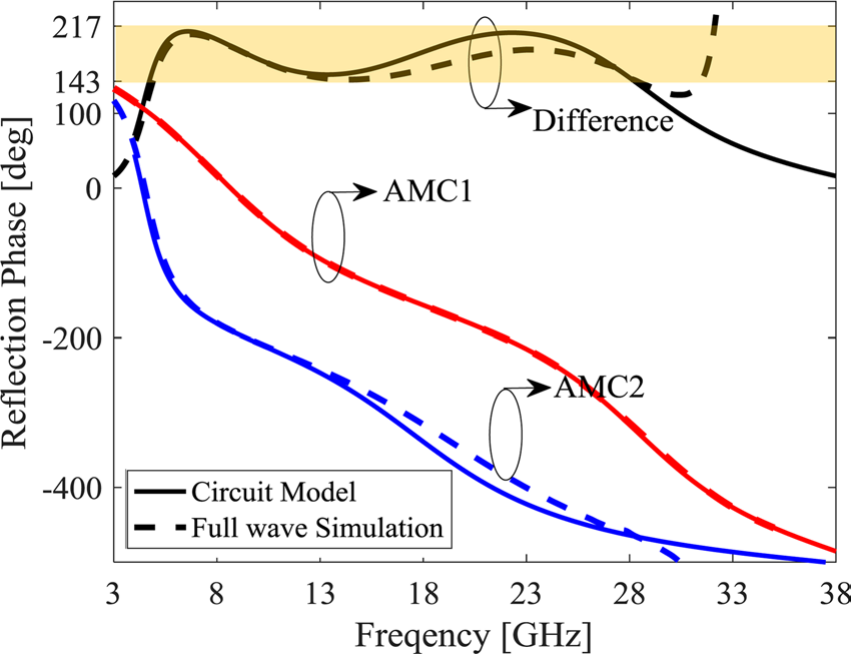
Reflection phase from two MAMC unit cells for low cost metasurface.

The commercially available FR-4 substrate is used in the third example to design low cost metasurface. In fact, the Rogers 5880 layer in the ROGERS metasurface is replaced by low cost FR-4 substrate and air gap where the air gap height is optimized to achieve closest effective ε_r_ to Rogers 5880 layer. Moreover, the lower patch which has air gap substrate should be carried out on a very thin substrate. Therefore, the both unit cell layers are composed of air gap and thin FR-4 substrate. The FR-4 substrates height which includes the patches are considered as 0.5 mm for both layers. The GA achieves optimized design values where air gap thickness is 3.9 mm and 1.23 mm for the lower and upper layers, a_1_ = 0.4 mm and a_2_ = 0.4 mm for MAMC1 and a_1_ = 7.29 mm and a_2_ = 2.09 mm for MAMC2. Similar to the second case, the unit cell periodicity is slightly tuned resulted in a = 8 mm. The desired phase difference (180° ± 37°) is achieved from 4.725 GHz to 28.24 GHz (142.6% fractional bandwidth) in the circuit model and 4.89 GHz to 28.37 GHz in the full wave simulation, corresponding to a 141% fractional bandwidth as shown in Fig. 8.

### Fabrication and test

**Figure 9:**
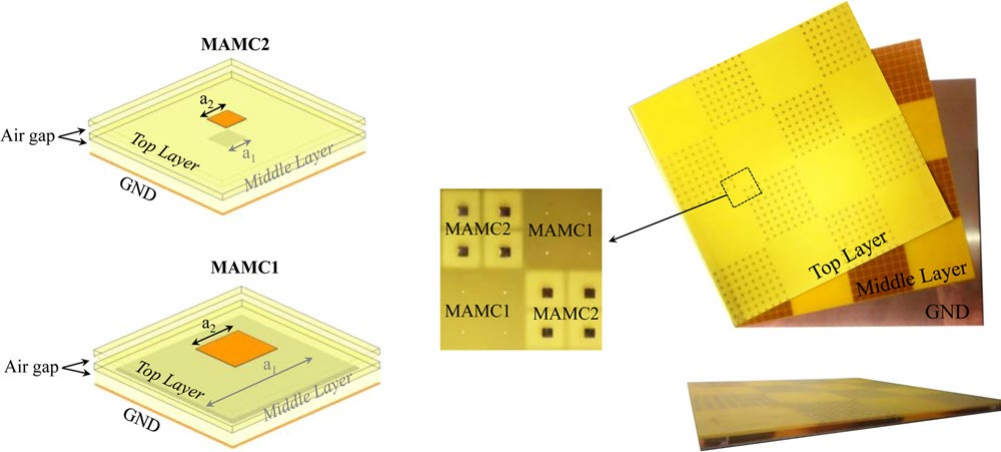
The fabricated low cost RCSR metasurface Measurement.

The third design example (low cost metasurface) is simulated and fabricated to demonstrate the idea. For this purpose, a checkerboard-like metasurface is formed by 4 × 4 alternating tiles where each tile consists of 8 × 8 identical unit cells as shown in Fig. 9. Considering that the size of each unit cell (8 mm × 8 mm), the overall dimensions of the metasurface is 256 mm × 256 mm. The 0.5 mm FR-4 lower layer is mounted on the copper sheet (GND) by using 3.9 mm thin Teflon rings spacers, while the top 0.5 mm FR-4 layer is suspended on the lower layer by 1.2 mm spacer as depicted in the figure.

**Figure 10:**
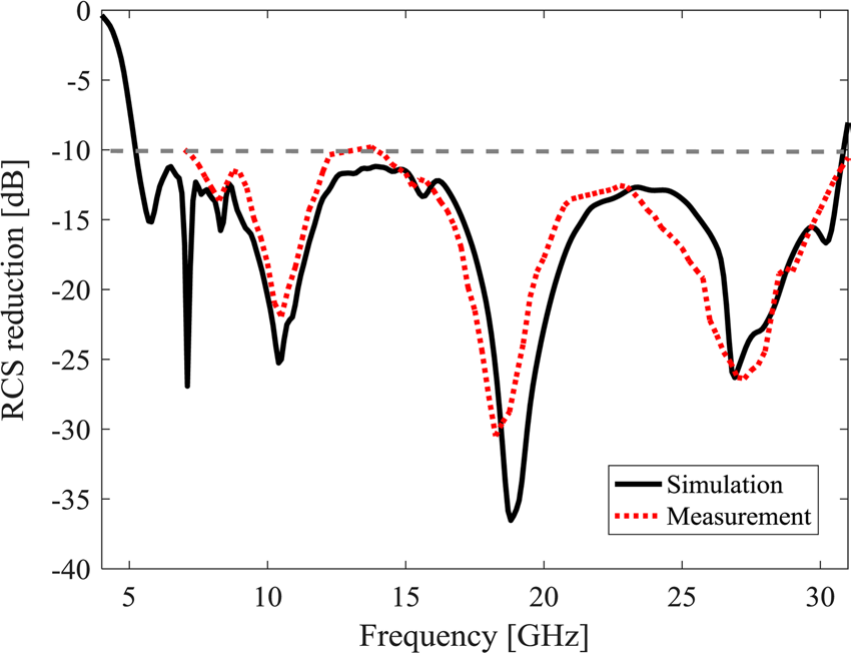
The simulation and measurement monostatic RCS reduction of the proposed low cost metasurface.

The surface RCSR is calculated from the metasurface RCS normalized to the RCS of a metal plate with the same physical dimension. Figure 10 depicts simulated and measured monostatic RCS reduction of the proposed surface at normal incident wave versus frequency. It is observed that 10-dB bandwidth RCS reduction is achieved from 5.22 GHz to 30.85 GHz, corresponding to a 142% 10-dB RCSR bandwidth. Notice that the simulation results are slightly different from the ones achieved in Sec.III (c) which is due to the mutual effect of tiles. Since the low cost available commercial off the shelf (COST) dielectric material is used to realize the air gap spacer, some small differences between simulated and measured results are appeared in Fig. 10.

**Figure 11:**
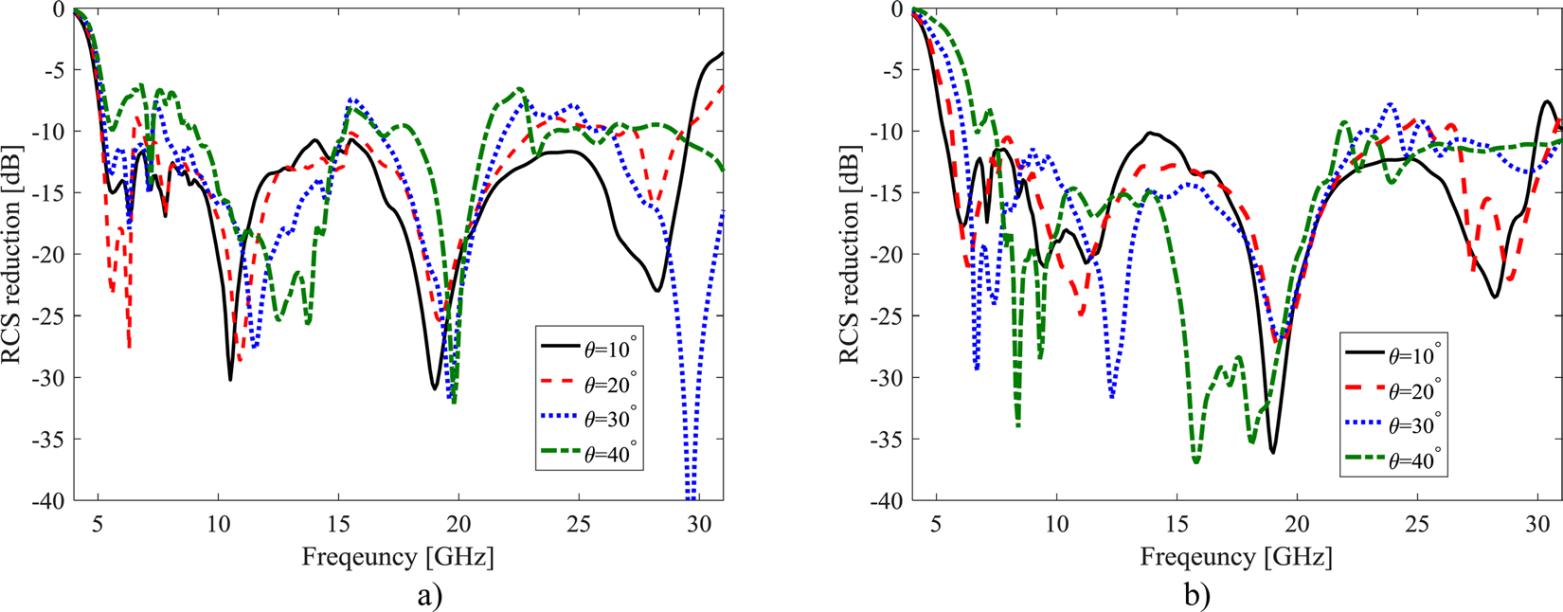
The RCS reduction of the proposed low cost metasurface for different incident angles, (**a**)TE- polarization (**b**) TM-polarization.

Figure 11 depicts the designed low cost metasurface performances in different oblique incident angles at both TE- and TM-polarization up to 40°. The 10-dB RCS reduction bandwidth of the surface are listed in Table 1 for different incident angles where the RCS reduction bandwidth is more than 98% for TM- and 51% for TE-polarizations up to 40° oblique incident angles.

**Table 1:** The 10-dB RCS reduction bandwidths for different incident angles.

Incident angle (θ°)	10-dB RCS reduction bandwidths	
	TM- Polarization	TE-Polarization
10	140	140.4
20	124	108.2
30	114.5	65.2
40	98	51

**Figure 12:**
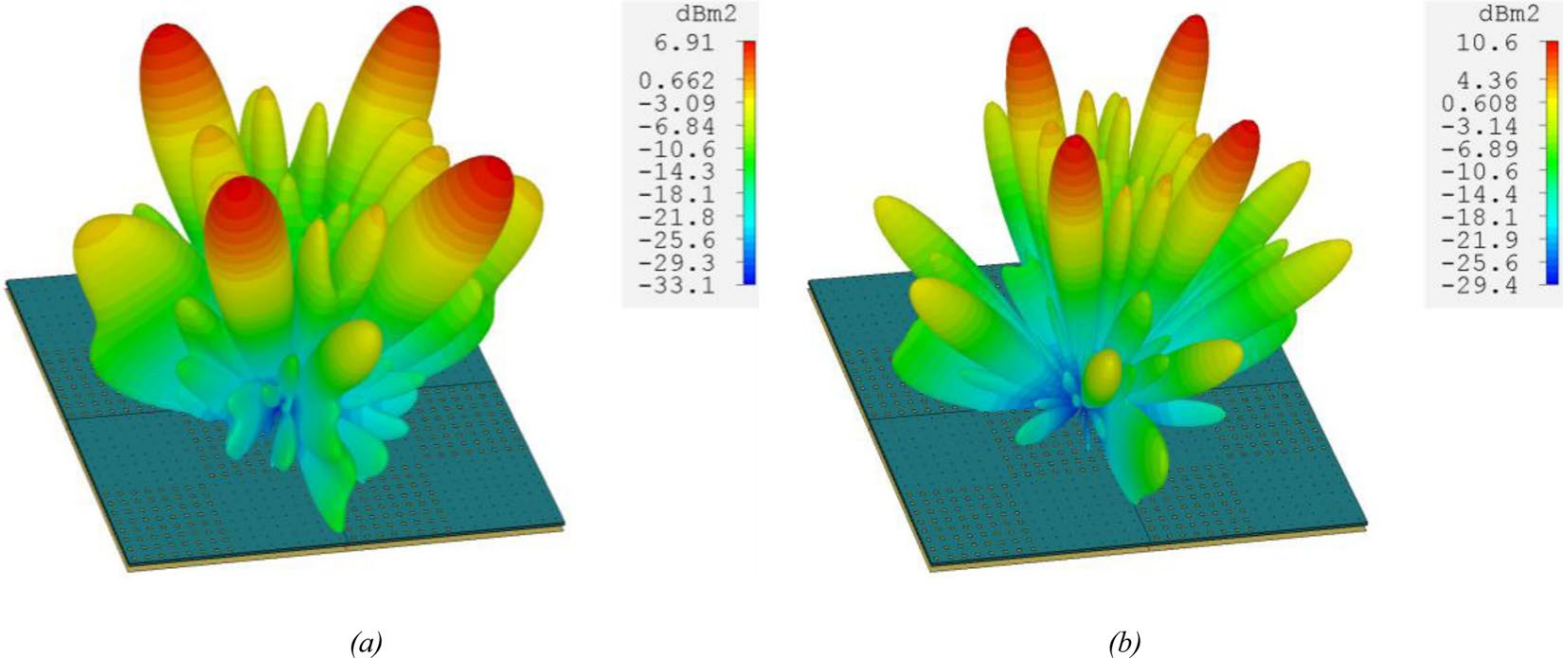
The 3D scatter radiation fields from the low cost metasurface at (**a**) 7.1 GHz (**b**) 10.4 GHz.

The 3D scatter radiation fields from the proposed metasurface are shown in Fig. 12 for normal incident wave at 7.1 GHz and 10.4 GHz which have the maximum RCS reduction values based on the results presented in Fig. 10. Under normal plane wave in Transient Solver of CST Microwave Studio, the reflected wave is mainly scattered in the diagonal planes (45° and 135°) due to the phase cancellation between adjacent tiles. Hence the monostatic RCS is minimum at the normal plane wave. Moreover, the scattering pattern of the chessboard layout has four main lobes and zeros in the incident angle.

Table 2 compares the proposed metasurface specifications with the state of the art researches. It is found that the RCS reduction bandwidth of this structure is significantly wider than the other works. In more details, the proposed structure achieves more than 142% RCS reduction bandwidth which has significantly wider rather than the best previous surface^7^. In addition, the start frequency of RCS reduction is low compared to the other works. Wide RCS reduction bandwidth, low cost and general design method are the main advantages of this work which proves its capability for practical applications.

**Table 2:** Comparison of the proposed low cost metasurface with state of the art references.

Structure	Thickness (mm)	10-dB Bandwidth		Substrate
		Normal incident (%)/Frequency range (GHz)	Oblique incident/ (%)	
^6^	6	122.3/6.2–25.7	—	F4B-2
^7^	2.52	126.5/9–40	—	FR-4
^9^	3	89/7.9–20.8	—	F4B
^10^	3	73/7.2–15.6	—	F4B
^13^	6.80	95/3.8–10.7	70% (40°)	RO4003 and PTFE
^14^	2.5	108/13.6–45.5	108% (50°)	FR-4
^4^	6.35	91/ 3.75–10 GHz	31% (40°)	RT/duroid-5880
^11^	2.524	109/ 13.1–44.5	93.5% (50°)	FR-4
^15^	11.508	91.5/3.77–10.14	90% (10°)	Rogers RO4350B
This paper	6.1	141.8/5.22–30.85 GHz	98% (40°)	FR-4

## Conclusion

In this paper, the non-resonant unit cells were designed, fabricated and measured for ultra-wideband RCSR. The proposed tile composed of a stack of non-resonant patches separated from one another by thin dielectric substrates and the whole structure is backed with a ground plane. The GA algorithm is adopted to optimize the geometry parameters of unitcell and find the best value. It was demonstrated that the 180° ± 37° reflection phase difference between two unitcell is achieved from 5.37–37.5 GHz corresponding to a 150% fractional bandwidth for *ideal model*, 6.37–31.72 GHz (133%) for *Rogers metasurface* substrate and 4.89–28.37 GHz (141%) for *low cost metasurface*. The *low cost metasurface* fabricated and measured which demonstrates wideband RCS reduction larger than 10 dB from 5.22–30.85 GHz (142%) for normal incidence plane wave. Compared to the references, this paper exhibits significantly wider RCSR bandwidth by using simple, thin and low cost structure.
